# Do We Need to Clamp the Renal Hilum Liberally during the Initial Phase of the Learning Curve of Robot-Assisted Nephron-Sparing Surgery?

**DOI:** 10.1155/2014/498917

**Published:** 2014-02-11

**Authors:** Ömer Acar, Tarık Esen, Ahmet Musaoğlu, Metin Vural

**Affiliations:** ^1^Department of Urology, VKF American Hospital, 34365 Istanbul, Turkey; ^2^School of Medicine, Koc University, 34450 Istanbul, Turkey; ^3^Department of Radiology, VKF American Hospital, 34365 Istanbul, Turkey

## Abstract

*Objective*. We aimed to compare the results of our initial robot-assisted nephron-sparing surgeries (RANSS) performed with or without hilar clamping. *Material and Method*. Charts of the initial RANSSs (*n* = 44), which were performed by a single surgeon, were retrospectively reviewed. R.E.N.A.L. nephrometry system, modified Clavien classification, and M.D.R.D. equation were used to record tumoral complexity, complications, and estimated glomerular filtration rate (eGFR), respectively. Outcomes of the clamped (group 1, *n* = 14) versus off-clamp (group 2, *n* = 30) RANSSs were compared. *Results*. The difference between the two groups was insignificant regarding mean patient age, mean tumor size, and mean R.E.N.A.L. nephrometry score. Mean operative time, mean estimated blood loss amount, and mean length of hospitalization were similar between groups. A total of 4 patients in each group suffered 11 Clavien grade ≥2 complications early postoperatively. Open conversion rates were similar. The difference between the 2 groups in terms of the mean postoperative change in eGFR was insignificant. We did not encounter any local recurrence after a mean follow-up of 18.9 months. *Conclusions*. Creating warm-ischemic conditions during RANSS should not be a liberal decision, even in the initial phases of the learning curve for a highly experienced open surgeon.

## 1. Introduction and Objectives

Nephron-sparing surgery (NSS) has recently emerged as a viable alternative for small renal tumours in patients with a normal contralateral kidney, with encouraging long-term oncological outcomes [1, 2]. Current guidelines advocate NSS as the ideal treatment of T1a tumors and an alternative treatment option for T1b tumors [3].

The utility of NSS has increased accordingly at many high volume centers over the past decade, approaching 90% for T1a tumors at some centers [4]. The more popular trend has been to perform NSS by minimally invasive approaches, which is a considerable challenge given concern about margin status and ischemia times [5, 6]. Robot-assisted nephron-sparing surgery (RANSS) overcomes a number of the technical hurdles of laparoscopic nephron-sparing surgery (LNSS) with a shorter learning curve. The use of robotics may facilitate minimally invasive NSS, and now it is accepted as an attractive minimally invasive treatment option for amenable tumors.

Considering the potential impact of warm-ischemia time (WIT) on renal functional outcomes after NSS, many techniques that reduce or eliminate WIT have been studied such as segmental parenchymal clamping [7], early unclamping of the renal hilum [8], and superselective clamping of the tumor-feeding vessels [9]. Performing the nephron-sparing procedure without obscuring the arterial inflow (off-clamp) is another option that serves to minimize renal ischemia and hence preserve renal function as much as possible. However, off-clamp procedures may be challenged when the visualization is compromised by suboptimal hemostasis and therefore there is a risk of surgical margin positivity and significant blood loss. Off-clamp NSS in a minimally invasive setting may further complicate the whole picture, particularly if you have just commenced doing robot-assisted laparoscopic surgery.

In this study, we aimed to present our institutional experience involving the initial RANSSs performed with or without renal hilar clamping. We particularly sought to evaluate the safety and feasibility of off-clamp RANSS, in the hands of an experienced open surgeon, during the initial phase of the learning curve.

## 2. Materials and Methods

A retrospective chart review identified 44 patients who have undergone RANSS after the adaptation of robotic technology in our institution, as of May 2010. All cases were performed by a single surgeon (TE) who is highly proficient in open NSS. Noteworthy, he did not have any laparoscopic surgery experience and he has accomplished a direct transition from open to robot-assisted surgery. Based on the status of renal perfusion during tumor excision, 2 subgroups were created. Group 1 consisted of the patients (*n* = 14) whose tumors were removed after clamping the renal hilum and the second group (*n* = 30) involved those who were managed with off-clamp RANSS.

**Figure 1:**
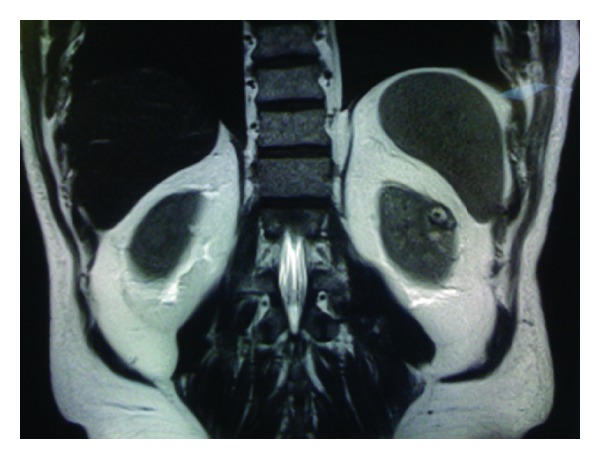
Coronal MR image of a 51-year-old male patient who has presented due to an incidentally diagnosed left renal mass. Tumor was measuring 2 cm in its greatest dimension and R.E.N.A.L. score was 8. He was managed by off-clamp RANSS. Eventual histopathologic diagnosis was Fuhrman grade 3, pT1a, and papillary RCC.

**Figure 2:**
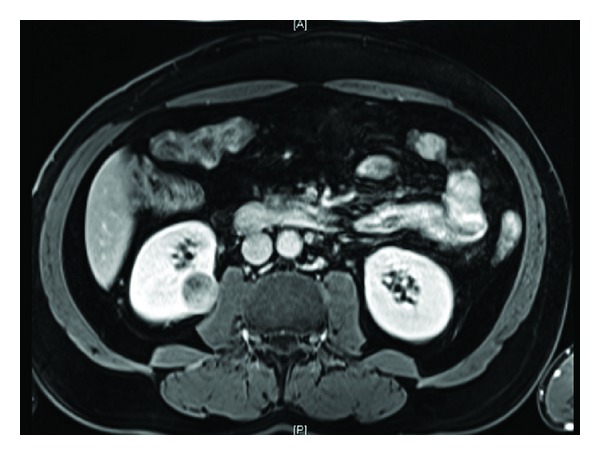
Axial MR image of a 56-year-old male patient who has presented due to an incidentally diagnosed right renal mass. Tumor was measuring 2.8 cm in its greatest dimension and R.E.N.A.L. score was 8. He was managed by off-clamp RANSS. Eventual histopathologic diagnosis was Fuhrman grade 3, pT1a, and papillary RCC.

**Figure 3:**
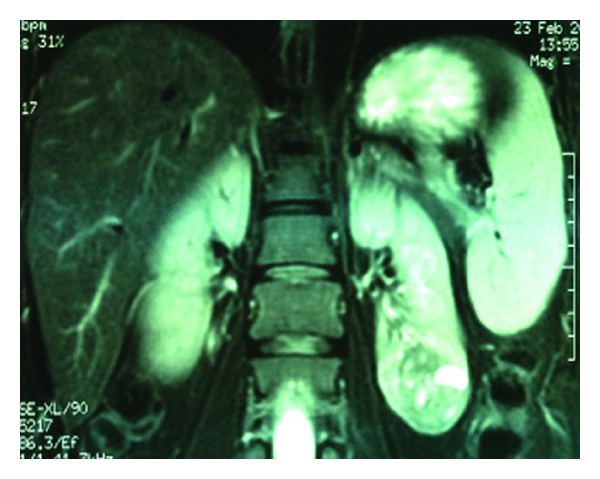
Coronal MR image of a 38-year-old female patient with an incidentally diagnosed left renal mass. Tumor was measuring 4.2 cm in its greatest dimension and R.E.N.A.L. score was 9. She underwent clamped RANSS. Eventual histopathologic diagnosis was Fuhrman grade 3, pT1a, and chromophobic RCC.

**Figure 4:**
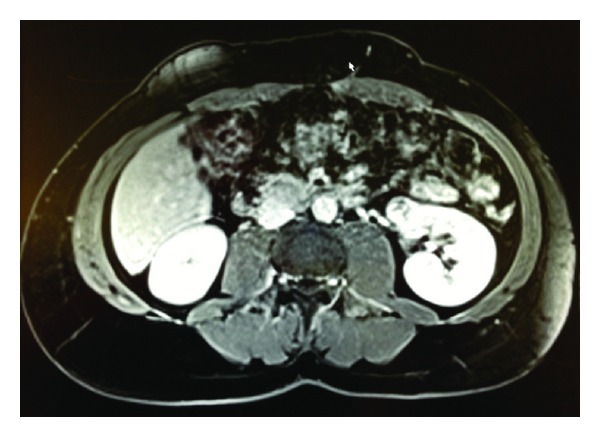
Axial MR image of a 34-year-old male who presented due to an incidentally diagnosed left renal enhancing mass. Maximal tumor diameter was 2.5 cm and R.E.N.A.L. score was 7. He was managed by clamped RANSS. Histopathologic examination of the excised specimen revealed Fuhrman grade 3, pT1a, and clear cell RCC.

All patients demonstrated contrast-enhancing renal masses on preoperative computed tomography and/or magnetic resonance imaging (Figures 1, 2, 3, and 4). All treatment options were discussed thoroughly with the patients. Indications for RANSS and selection of the off-clamp technique were based on tumor characteristics (size, complexity, and location), patient comorbidities, and surgeon preference.

Patient characteristics were analyzed including demographic data, past medical history, mode of presentation, comorbidities, and American Society of Anesthesiologists' (ASA) score. Radiologic characteristics of the renal masses were scored by a senior urologist and a senior radiologist according to R.E.N.A.L. nephrometry system, as described elsewhere [10]. Tumor size was defined as the largest diameter of the tumor (cm). Based on preoperative radiologic findings, none of the patients had lymph node involvement or distant metastasis.

Operative data consisted of total operative time, estimated blood loss (EBL), and warm-ischemia time (WIT). All complications within 30 days of surgery were classified according to the modified Clavien classification system [11]. Serum creatinine was measured 1 day, 1 month, 3 months, and 6 months and then yearly after surgery. Estimated glomerular filtration rate (eGFR) was determined using the modified modification of diet in renal disease (MDRD) equation [12]. Preoperative eGFR was compared to eGFR on the last follow-up to assess long-term changes in renal function after RANSS. Pathological data included histological subtype, T stage, and margin status. Tumor staging was designated according to the TNM classification based on the 2009 American Joint Committee on Cancer/International Union against Cancer Classification System.

Tumor recurrence was defined as a new renal mass in the resection bed of the kidney in the absence of distant metastasis based on imaging. Follow-up was calculated from the time of surgery until the last known contact with the patient or until the date of death. Mortality status was confirmed with death certificates.

Statistical calculations were performed using the commercially available software (SPSS version 20). Continuous variables were compared using the paired *t*-test and categorical variables were compared with the chi-square test. Any *P* < 0.05 defined statistical significance.

### 2.1. Surgical Technique

Robot-assisted nephron-sparing surgeries were performed using the da Vinci Si HD surgical system (Intuitive Surgical, Inc., Sunnyvale, CA) with a 5-port approach, including two 8 mm ports for robotic instruments, one 12 mm port for the robotic scope, and two ports for the bedside assistant. RANSSs were carried out through the transperitoneal route with the patient in the flank position. After ensuring adequate renal exposure and sweeping the perinephritic fatty tissue away from the tumor, tumor margins were demarcated with electrocautery. Intraoperative ultrasonography was utilized in order to determine the location, boundary, and depth of endophytic lesions. In the clamped RANSS group, the renal artery and the vein were occluded together with an external vessel loop secured with a hem-o-lok clip over a silicone tube. In the off-clamp group, renal hilum was dissected to enable rapid hilar control in case of excessive bleeding during tumor excision. Tumors were enucleoresected with cold scissors, leaving a minimal rim of normal parenchyma. Bleeding vessels were controlled using monopolar or bipolar electrocautery. Large blood vessels at the resection bed and/or collecting system defects were closed using 3.0 polyglactin sutures. Renal parenchyma was reapproximated over bolsters using the “sliding clip” technique. Topical hemostatics were applied to the surface. Perinephritic fatty tissue was adapted over the surgical field to serve as a natural tamponade. Tumor bed was inspected under low insufflation pressure to ensure hemostasis after renal reconstruction. After extracting the specimen, gross margins were inspected before sending the specimen to pathology for permanent section.

## 3. Results

A total of 44 patients (33 male and 11 female) with a mean age of 49.59 (SD = 13.4) have undergone RANSS between May 2010 and July 2013.  Table 1 outlines patient characteristics, tumor characteristics, perioperative outcomes, and functional outcomes in patient subgroups. Mean patient ages were 46.2 (SD = 15.05) and 51.1 (SD = 12.5) years in groups 1 and 2, respectively (*P* = 0.26). Mean ASA score did not differ significantly between the 2 groups (1.2 (SD = 0.4) in group 1 versus 1.4 (SD = 0.5) in group 2, *P* = 0.26). Apart from a single patient in group 1, who had a tumor bearing solitary kidney, all nephron-sparing surgery decisions were based on elective indications. Majority of the patients (71.4% in group 1 and 76.6% in group 2) were diagnosed incidentally. All patients underwent RANSS for a single mass.

The difference between the two groups was insignificant regarding mean tumor size (3.6 (SD = 1.4) in group 1 versus 3.8 (SD = 3.1) in group 2, *P* = 0.88). Although tumors operated under warm ischemia had higher mean R.E.N.A.L. (6.6 (SD = 1.9) versus 5.9 (SD = 1.8)), P.A.D.U.A scores (7.8 (SD = 1.6) versus 7.2 (SD = 1.6)), and lower mean C-index value (1.4 (SD = 0.4) versus 1.5 (SD = 0.4)), the difference between the two groups remained statistically insignificant in terms of tumor morphometry.

Mean operative duration was nonsignificantly longer in group 1 (149.2 (SD = 41.04) versus 142.3 (SD = 54.8) minutes, *P* = 0.67). Mean warm-ischemia time was 22.5 (SD = 6.2) minutes in group 1. Although the difference was statistically insignificant, mean estimated blood loss amount was higher in group 2 (170.3 mL (SD = 80.5) in group 1 versus 201.6 mL (SD = 179.3) in group 2, *P* = 0.53).

Table 2 depicts the pathologic outcome. Surgical margins did not harbor malignant infiltration in any case. The most common histopathologic diagnosis was clear cell renal cell carcinoma (64.2% in group 1, 36.6% in group 2). Four patients in group 1 (28.5%) and 4 patients (13.3%) in group 2 had tumors larger than 4 cm but confined to the kidney (pT1b and pT2).

Two patients in group 1 and 3 patients in group 2 required blood transfusion postoperatively. A total of 4 patients in each group suffered 11 Clavien grade ≥2 complications within 30 days of surgery. Apart from transfusions, one patient in group 1 needed cystoscopic clot evacuation, double-j insertion, and angioembolization due to persistent postoperative hemorrhage. Double-j insertion due to urinary extravasation (*n* = 1) and paralytic ileus (*n* = 1) were the other recorded complications in this group. For group 2, paralytic ileus (*n* = 1) and angiographic embolization of a bleeding pseudoaneurysm (*n* = 1) represented the complications that were recorded in addition to the blood transfusions. Open conversion rates did not differ significantly (1/14 in group 1 versus 4/30 in group 2) between groups.

Preoperative eGFR was 92.4 mL/min/1.73 m^2^ (SD = 18.5) and 90.6 mL/min/1.73 m^2^ (SD = 16.4) in groups 1 and 2, respectively (*P* = 0.74). At a mean follow-up duration of 18.9 months (SD = 10.1), mean eGFR in group 1 was 88.2 mL/min/1.73 m^2^ (SD = 21.7), compared to 80.6 mL/min/1.73 m^2^ (SD = 16.4) preoperatively (*P* = 0.209). The difference between the 2 groups in terms of the mean postoperative decrease in eGFR was not significant (6.7 (SD = 6.8) versus 10.8 (SD = 8.9) mL/min/1.73 m^2^ in group 1 and 2, resp., *P* = 0.13). None of the patients in this cohort required any form of renal replacement therapy during the postoperative period. We did not encounter any local recurrence or mortality during follow-up.

## 4. Discussion

The impact of WIT on renal functional preservation after NSS is still a matter of debate. The “safe” duration of WIT, after which complete recovery of renal function could be expected, was once commonly considered to be 30 minutes [13, 14]. In view of studies demonstrating negligible effects on renal function after renal pedicle clamping for 90 minutes in porcine models [15–17] as well as retrospective clinical observations suggesting that WIT of 40 to 55 minutes may be tolerated [18], some have suggested that the kidney can tolerate longer periods of WIT. Conversely, others have defined the safe WIT to be 20 minutes [19–21]. In the landmark study, conducted by a conjoint team from Cleveland and Mayo Clinic, the impact of WIT as a continuous variable on renal functional outcomes has been investigated on 362 patients with a solitary kidney undergoing open or laparoscopic PN. The main finding of this study was that longer WIT was associated with increased odds of acute renal failure as well as development of new-onset stage IV chronic kidney disease during follow-up. Thus, the authors concluded that “every minute counts when the renal hilum is clamped without hypothermic techniques” [22].

Based on these observations, various refinements in surgical technique aimed at minimizing or eliminating WIT have been described. “Unclamping technique,” whereby the hilar vessels are clamped only during sharp dissection of the tumor, closure of exposed vessels, and repair of collecting system defects at the resection base, was innovated and employed by Gill et al. [8]. They were able to decrease their WIT for LNSS from 31.6 to 14.4 minutes using this technical modification [23]. “Selective renal parenchymal clamping,” which denotes regional clamping of the renal parenchyma only in the area of planned excision, has been used by Viprakasit et al. who have reported successful results with this technique in three patients undergoing RANSS [7]. Gill et al. described a novel technique in which tumor excision is performed without hilar clamping in the setting of medically induced and carefully monitored hypotension [24]. The authors reported successful results using this technique in 12 cases of LNSS and 3 cases of RANSS [24]. Recently, the same group has developed another technique, which consists of anatomical vascular microdissection and preemptive control of tumor specific, tertiary, or higher order renal arterial branch(es) using neurosurgical aneurysm microbulldog clamps which is made possible after gathering detailed anatomic information about their location and course within the renal parenchyma via 3D reconstruction of cross-sectional images [9]. In this study, out of 58 cases undergoing anatomical zero-ischemia NSS, 15 were operated via the robot-assisted laparoscopic route. Mean tumor size and mean R.E.N.A.L. score were 3.2 cm and 7 in this series. They did not report any intraoperative complications and the majority of postoperative complications were of low grade. None of the patients had surgical margin positivity. Of note, 21% of the patients received a perioperative blood transfusion however none had suffered an acute or delayed renal hemorrhage [9].

Another potential strategy for reducing warm-ischemic injury to the kidney is the selective application of NSS under completely perfused conditions, whereby vasculatures of both the renal unit and the tumor remain unobscured throughout the whole operation. Although such “off-clamp” procedures may improve preservation of renal function [25], excision and reconstruction of the kidney may be quite troublesome on a bloody surgical field. This technique has been reported only to a limited degree in the minimally invasive surgery literature with surgeons using ablative energy or enucleation only to make such resections possible, or limiting off-clamp resections to very superficial tumors [26–29].

White et al. reported the first such series of RANSS without renal hilar occlusion in 8 patients. They denied using the technique for patients with endophytic, hilar, or multiple tumors, tumors >4 cm, or patients with a solitary kidney and they used the harmonic scalpel for all resections [30]. Several other authors have confirmed the safety and feasibility of off-clamp RANSS [31, 32], even for complex renal tumors [33]. Recently, Sandhu et al. reported their off-clamp RANSS experience which included 47 tumors in 39 patients. Mean operative time and mean estimated blood loss were 147 minutes and 219 mL, respectively. They did not report any intraoperative complication or surgical margin positivity. They commented in favor of the off-clamp RANSS technique in experienced hands [34]. In the multi-institutional study that has been conducted by Kaczmarek et al., perioperative results of 49 patients who had undergone off-clamp RANSS (mean tumor size: 2.5 cm, mean R.E.N.A.L. nephrometry score: 5.3) were compared against propensity score matched clamped controls. Off-clamp RANSS patients had a significantly shorter mean operative time, a higher EBL, and a smaller decrease in eGFR than their clamped counterparts. They highlighted the safety and feasibility of off-clamp RANSS in appropriately selected patients and with adequate surgeon experience [31].

In this study, we evaluated our institutional experience that involves the initial off-clamp and clamped RANSSs. The operating surgeon in our series has made use of his extensive open NSS experience during the transition period from open to robot-assisted surgery. Although he has never been a pure laparoscopic surgeon, the skills gained through many years of open surgery enabled him not to be reluctant to perform off-clamp NSS in the beginning of his robotic career. Thereby, he was able to implement what he did as an open surgeon directly to robotic platform.

Patient characteristics were similar between study groups. Majority of our patients were below 65 years of age (37/44, 84%) and all except one patient in the off-clamp group had ASA scores of either 1 or 2. Mean tumor size was marginally and insignificantly higher in the off-clamp group. A substantial portion of the excised tumors was measuring <4 cm (33/44, 75%) with 4 patients in each group having ≥pT1b disease. Morphometric scores were denoted towards a relatively more complicated tumor profile in the clamped group; however, the difference was again lacking statistical significance. The majority (11/14, 78.5%) of the tumors in the clamped group were of moderate or severe complexity. On the other hand, 66.6% (20/30) of the tumors in the off-clamp group had a R.E.N.A.L. score between 4 and 6. These findings might limit the reproducibility of our findings, since we seem to be dealing with “healthier” patients and “easier” tumors. Moreover, more complicated tumors have been resected in a relatively “bloodless” field. However, these were consecutive patients and the majority of them were managed by off-clamp NSS.

Undoubtedly, hemorrhagic complications are an anticipated issue while doing off-clamp NSS. In our study, there was quantitatively more blood loss in the off-clamp group. However, the amount was not significantly different than that calculated in the clamped group and almost the same number of patients (2 in group 1 versus 3 in group 2) received blood transfusions postoperatively in both groups. Leaving a minimal parenchymal margin on the tumor instead of doing deeper resections, performing a 2-layer (deep layer and capsular layer) closure, interposing bolsters that enable direct compression on the tumor bed, utilizing topical hemostatics, and adapting fatty tissue over the surgical field may all help us to minimize the amount of blood loss, especially in the off-clamp group. Putting blood transfusions aside, the number of high grade (Clavien grade 3–5) complications was also similar between groups. Bleeding from the tumor bed might hinder proper visualization during NSS and trying to stay as close as possible to the pseudocapsule while doing enucleoresection may both lead to surgical margin positivity. However, this was not the fact in our series and none of the patients in either group had tumoral infiltration in their surgical margins.

Despite the fact that our follow-up period is relatively short (18.9 ± 10.1, range = 1–36), functional results were not significantly different from each other when groups were compared. The mean drop in eGFR was marginally higher in the off-clamp group (*P* = 0.13) which may partly be explained by the relatively higher mean ASA score in this group of patients.

Our data reflects the period of transition from open to robot-assisted surgery. Numbers are small in the study groups and we have a relatively limited duration of follow-up. Retrospective study design with its inherent biases is another limitation of our study. Another criticism is the lack of strictly defined indications for off-clamp RANSS. Size, location, and complexity of the tumor were the main determinants of the decision to clamp or not to clamp renal hilum. General health, comorbidity status of the patient, and surgeon preference were the other factors that were considered before making this decision. Nevertheless, selection bias should be taken into account while interpreting our results.

Our preliminary findings have demonstrated that off-clamp RANSS can be a safe and feasible option in the hands of an experienced open surgeon even in the initial phase of the learning curve. However, our results may not necessarily be applicable to larger tumors with a more complicated morphometric profile.

## 5. Conclusions

Perioperative, functional, and oncologic outcomes of the initial off-clamp RANSSs were similar to that of RANSSs performed under warm-ischemic conditions. Clamping the renal hilum during RANSS should not be a liberal decision, even in the initial phase of the learning curve for a highly experienced open surgeon. Further studies are needed to compare the outcomes of off-clamp RANSS with those of the traditional clamped RANSS to assess the relative efficacy and feasibility of the off-clamp technique.

## Figures and Tables

**Table 1:** Differences between group 1 and group 2 in terms of patient characteristics, tumor characteristics, perioperative results, and functional results.

	Group 1 (*n* = 14)	Group 2 (*n* = 30)	*P* value
Patient characteristics			
Mean age, years (SD)	46.2 (15.05)	51.1 (12.5)	0.26
Mean ASA score (SD)	1.2 (0.4)	1.4 (0.5)	0.26
Male, *n* (%)	11 (78.5)	22 (73.3)	1
Tumor characteristics			
Mean size, cm (SD)	3.6 (1.4)	3.8 (3.1)	0.88
Mean R.E.N.A.L. score (SD)	6.6 (1.9)	5.9 (1.8)	0.25
Right-sided tumors (%)	3/14 (21.4)	15/30 (50)	0.1
Perioperative results			
Mean operative time, min (SD)	149.2 (41.04)	142.3 (54.8)	0.67
Mean WIT, minutes (SD)	22.5 (6.2)	0	—
Mean EBL, mL (SD)	170.3 (80.5)	201.6 (179.3)	0.53
Mean length of stay, days (SD)	4.2 (1.6)	3.8 (1.1)	0.32
Intraoperative complications, *n* (%)	0 (0)	0 (0)	—
Clavien grade ≥ 2 complications, *n* (%)	4 (28.5)	4 (13.3)	0.2
Conversions to open, *n* (%)	1 (7.1)	4 (13.3)	1
Functional results			
Mean preoperative eGFR, mL/min/1.73 m^2^ (SD)	92.4 (18.5)	90.6 (16.4)	0.74
Mean eGFR at last control, mL/min/1.73 m^2^ (SD)	88.2 (21.7)	80.6 (16.4)	0.2
Mean change in eGFR, mL/min/1.73 m^2^ (SD)	6.7 (6.8)	10.8 (8.9)	0.13

SD: standard deviation.

**Table 2:** Differences between group 1 and group 2 in terms of histopathologic variables.

	Group 1 (*n* = 14)	Group 2 (*n* = 30)
Tumor pathology		
Clear cell RCC, *n* (%)	9 (64.2)	11 (36.6)
Papillary RCC, *n* (%)	1 (7.1)	3 (10)
Chromophobe RCC, *n* (%)	2 (14.2)	5 (16.6)
Oncocytoma, *n* (%)	0 (0)	4 (13.3)
Angiomyolipoma, *n* (%)	1 (7.1)	3 (10)
Others, *n* (%)	1 (7.1)	4 (13.3)
Tumor T stage		
T1a, *n* (%)	8 (57.1)	15 (50)
T1b, *n* (%)	3 (21.4)	3 (10)
T2, *n* (%)	1 (7.1)	1 (3.3)
Benign, *n* (%)	2 (14.2)	11 (36.6)

Positive surgical margins, *n* (%)	0 (0)	0 (0)
